# Incidence of *Fusarium* Mycotoxins in Wheat and Maize from Albania

**DOI:** 10.3390/molecules26010172

**Published:** 2020-12-31

**Authors:** Dritan Topi, Janja Babič, Katarina Pavšič-Vrtač, Gabrijela Tavčar-Kalcher, Breda Jakovac-Strajn

**Affiliations:** 1 Institute of Food Safety, Feed and Environment, Veterinary Faculty, University of Ljubljana, Gerbičeva 60, 1000 Ljubljana, Slovenia; dritan.topi@unitir.edu.al (D.T.); janja.babic@vf.uni-lj.si (J.B.); katarina.vrtac@vf.uni-lj.si (K.P.-V.); breda.jakovacstrajn@vf.uni-lj.si (B.J.-S.); 2 Department of Chemistry, Faculty of Natural Sciences, University of Tirana, Boulevard Zogu 1, 25, 1016 Tirana, Albania

**Keywords:** *Fusarium* toxins, deoxynivalenol, fumonisins, maize, wheat, Albania

## Abstract

In this study, ten *Fusarium* toxins were analysed in wheat and maize commodities from Albania. In total, 71 samples of wheat and 45 samples of maize were collected from different producing regions. The analytical procedure consisted of a simple one-step sample extraction followed by the determination of toxins using liquid chromatography coupled with tandem mass spectrometry. *Fusarium* toxins were found in 23% of the analysed wheat samples and in 78% of maize samples. In maize samples, most often fumonisins B_1_ (FB1) and B_2_ (FB2) were found. They were present in 76% of samples. They were detected in all positive samples except in one with concentrations ranging from 59.9 to 16,970 μg/kg. The sum of FB1 and FB2 exceeded the EU maximum permitted level (4000 μg/kg) in 31% of maize samples. In wheat samples, the only detected *Fusarium* mycotoxin was deoxynivalenol (DON), present in 23% of samples. In one sample with the concentration of 1916 μg/kg, the EU maximum permitted level (1250 μg/kg) was exceeded. This is the first report on the presence of *Fusarium* toxins in wheat and maize grains cultivated in Albania.

## 1. Introduction

Mycotoxins are secondary metabolites with low molecular weight produced by filamentous fungi, or more specifically, moulds [1,2,3,4,5,6,7,8]. They contaminate cereals and other foods like nuts, spices, fruits, and their by-products [3,7]. In crops, they can be formed before harvesting, during harvest, drying, and storage in non-appropriate conditions [3,6,7,8,9,10,11]. They are mainly produced by the species *Aspergillus*, *Penicillium*, *Alternaria*, *Claviceps*, and *Fusarium* [3,9,11,12,13,14,15]. *Fusarium* species are probably the most dominant toxin-producing fungi in the temperate regions of the Northern hemisphere, responsible for *Fusarium* head blight disease in small grain cereals and ear rot disease in maize that may cause severe reductions in crop yield [4,14,16,17]. The main *Fusarium* toxins are type A and type B trichothecenes, zearalenone (ZEA), and fumonisins (FBs) [4,12,18]. The Type-A group includes T-2 toxin, HT-2 toxin, neosolaniol (NEO), and 4,15-diacetoxyscirpenol (DAS), while the Type-B group includes nivalenol (NIV), deoxynivalenol (DON), its 3-acetyl (3-AcDON) and 15-acetyl (15-AcDON) derivatives, and fusarenone-X [16].

Mycotoxins are relatively stable, and prevention methods to prevent mycotoxin contamination on the field before harvest, during harvesting, or storage have been developed, while their complete elimination from the contaminated material is difficult if not impossible [6,7,8,9,10,16,17,19,20]. However, sorting, cleaning, debranning, and thermal processing can significantly reduce mycotoxin concentrations in cereal products, whereas milling processes result in the repartitioning of mycotoxins in different milling fractions leading to a reduction in mycotoxin burden in the fractions used for human consumption [21,22]. The fate of different mycotoxins during these processes have been studied by several authors [22,23,24,25,26,27]. However, the results presented by Griessler et al. [20] reporting high contamination levels of FBs in complementary and complete feeding stuff prove that mycotoxins burden the fractions commonly used as animal feed [21,22,26]. Mycotoxin contamination in different food and feed commodities is an ongoing global threat and will be more significant due to the climate changes and increased exchanges in the food and feed global market [11,15,19]. Exposure to these compounds is a significant threat to human and animal health and is associated with different acute or chronic mycotoxicoses [1,6,7,9,15,16,19,28].

In the European Union, the presence of *Fusarium* toxins in foodstuffs is regulated by Regulation (EC) No 1126/2007 [29] amending Regulation (EC) No 1881/2006 [30], and by Recommendation 2013/165/EU [31].

Several surveys on the worldwide contamination of *Fusarium* toxins in cereals and cereal products during the last decade indicate the importance of the problem that food safety is facing globally [3,4,5,19,28,32]. Several papers presented data on the *Fusarium* mycotoxins occurrence from European countries [9,18,20,33,34,35,36,37,38,39,40,41,42,43,44,45,46,47,48,49,50]. These data have shown high contamination rates, ranging from 20% to 100% for DON, 11% to 95% for FBs, from 1% to 100% for T-2, from 4% to 60% for HT-2 and from 71% to 100% for ZEA. However, the incidence of samples exceeding the EU maximum permitted levels was predominantly low. The authors often reported zero incidence or single samples exceeding maximum permitted levels [34,35,38,39,42,43,46]. Others, like Kirinčič et al. [47], reported only 2.4% of cereal and cereal product samples investigated in 2008–2012 in Slovenia and Van der Fels-Klerx et al. [41] reported 2.5% of wheat and 8.5% of oat and maize samples from countries of the Northern Europe investigated during the period 1989–2009 containing mycotoxins above the EU legal limits. Bryła et al. [48] reported 6.8% of cereal samples, cultivated in Poland in 2014, exceeding the maximum acceptable DON levels, and Udovicki et al. [9] reported 11.3% of samples from Serbia exceeding the EU maximum permitted levels during the period 2004–2016.

For the determination of mycotoxins, enzyme-linked immunosorbent assay (ELISA) [40,42,44,49], gas chromatography–mass spectrometry [35,38] and liquid chromatography coupled with ultraviolet [20,47], fluorescence [20,33,34,47] or mass spectrometry [33,37,39,43,45,46,47,48] detectors were used. High-performance liquid chromatography with fluorescence detection (HPLC–FLD) is most frequently used for the quantitative determination of single mycotoxins, whereas liquid chromatography coupled with mass spectrometry allows for the simultaneous determination of multiple mycotoxins [15,18,28].

This study aimed to get an insight into the occurrence of ten *Fusarium* mycotoxins (DON, 3-AcDON, 15-AcDON, ZEA, FB1, FB2, T-2, HT-2, DAS, and NEO) in two primary cereal commodities produced in Albania, maize, and wheat, both used as food and feed [51]. The study is the first-ever report on the presence of these mycotoxins in crop commodities from Albania. The data will expand the information on the occurrence of these mycotoxins in different grains from Southern Europe. We expected similar occurrence rates and concentrations as reported from the Southern European and Central European countries.

## 2. Results and Discussion

### 2.1. Occurrence of Fusarium Toxins in Samples

The samples containing one or more individual *Fusarium* toxins at concentrations above limit of quantification (LOQ) were considered as positive. Altogether, 44% of the analysed wheat and maize samples were contaminated. In the year 2014, the contamination rate was 58%, while in 2015 it was 26%.

In winter wheat samples from all regions, DON was the only detected *Fusarium* toxin, while the concentrations of the other nine *Fusarium* toxins were below LOQ (50 µg/kg). The number and the percentage of positive samples, the mean value of positive samples, the median, the minimum, and maximum determined concentrations are presented in Table 1.

The maximum permitted level of 1250 µg/kg given in Regulation (EC) No 1126/2007 [29] for unprocessed cereals other than durum wheat, oats, and maize intended for human consumption was exceeded in only one of the wheat samples from the year 2015 with a DON concentration of 1916 µg/kg.

The contamination rate in maize was much higher than in wheat. In Table 2, the incidence, mean value of positive samples, median, minimum, and maximum concentrations of *Fusarium* mycotoxins are presented for maize commodities. The main mycotoxins detected were FB1 and FB2. In the year 2015, they were the only mycotoxins found in maize. FB1 was present in all contaminated samples but one. In two-third of cases, both FB1 and FB2 were present, while in one-third of samples, only FB1 was detected. No sample contained only FB2. In the samples collected in 2014, DON, ZEA, and T-2 were also detected. ZEA was found in two samples from Kruja, and the T-2 toxin was detected in one sample from the Korça region. The results were in line with the global risk maps presented by Battilani and Logrieco [52]. According to the maps, Albania is among the countries with a low/absent risk regarding the DON contamination of wheat at harvest and among the countries with a high global risk of fumonisin contamination of maize. However, in the samples of barley and rye, no *Fusarium* toxins were detected.

Concerning the sum of FB1 and FB2, the maximum permitted level of 4000 µg/kg laid down in Regulation (EC) No 1126/2007 [29] for unprocessed maize was exceeded in 12 samples (39%) in 2014 and two samples (14%) in 2015, altogether in 31% of maize samples. In six samples from the year 2015, the concentration was even higher than 10,000 µg/kg. The concentrations of DON, ZEA, and T-2 toxin in maize samples were lower than the maximum permitted levels and the EU indicative level for the sum of T-2 and HT-2 in unprocessed maize intended for human consumption (1750 µg/kg, 320 µg/kg, and 200 µg/kg, respectively) given in Regulation (EC) No 1126/2007 and Commission Recommendation 2013/165/EU [29,31].

The data on the presence of DON in winter wheat and maize according to the regions are shown in Table 3 and Table 4, respectively. The incidence in wheat was similar in two western regions, Fieri and Lushnja, situated along the Adriatic Sea and the Elbasan region in the inner part of the country, but it was considerably lower in the eastern region Korça with the temperate continental climate (Table 3).

The concentrations were below the maximum permitted level of 1250 µg/kg, except in one sample from Elbasan, where the concentration was 1916 µg/kg. The incidence of DON in the maize varied from 0% (Fieri and Lushnja) to 64% (Kruja) (Table 4). All the concentrations were far below the maximum permitted level of 1750 µg/kg. However, the incidence and concentrations of FBs were higher. In Fieri, Lushnja, and Kruja, the mean concentrations (sum of FB1 and FB2) were higher than the maximum permitted level (4000 µg/kg). Concerning the median values of positive samples, the order of the regions was Fieri > Lushnja > Kruja > Elbasan > Korça, situated from the west of the country with a hot-summer Mediterranean climate to the east of the country belonging to the continental climate. However, the highest concentration was determined in a sample from Korça. As mentioned before, the maximum permitted concentration was exceeded in 14 samples. Most of these samples were from Lushnja and Kruja, where 50% and 36% of samples, respectively, were non-compliant. However, the incidence of contaminated maize samples containing at least one mycotoxin was similar in the Fieri, Lushnja, and Korça regions (57%, 70%, and 64%, respectively), while it was higher in the Kruja and Elbasan regions (91% and 100%, respectively).

The co-occurrence of two or more *Fusarium* toxins was detected only in maize samples. Of 34 contaminated samples, five samples (15%) contained one mycotoxin, 19 samples (56%) contained two mycotoxins, nine samples (26%) contained three mycotoxins, and one sample (3%) contained four mycotoxins. This finding is consistent with the results presented by Jakovac-Strajn et al. [34], Ibáñez-Vea et al. [38], Stanković et al. [40], Juan et al. [43], Alkadri et al. [46], and Kirinčič et al. [47], who reported a high percentage of samples containing more than one mycotoxin. FB1 and FB2 co-occurred most often. They were present together in 18 of the 19 samples containing two mycotoxins. While in eight of the nine samples containing three mycotoxins, DON, FB1, and FB2 co-occurred, in one sample, DON, ZEA, and FB1 were present. In the sample with four mycotoxins, DON, ZEA, FB1, and FB2 were present. However, the most common co-occurrence of DON, 3-AcDON, and 15-AcDON reported by Ibáñez-Vea et al. [38] and Van Der Fels-Klerx et al. [41] was not recognized in our study.

The incidence rate and the mean concentrations of mycotoxins were higher in the year 2014 than in 2015 in both commodities. However, the mycotoxin pattern was different between wheat and maize, which could be dependent on *Fusarium* species that infect these two cereals [4]. Both the production of mycotoxins and *Fusarium* profile are dependent on several factors, primarily climatic conditions, particularly rainfall and temperature at the flowering stage, but also agronomic factors, such as tillage, nitrogen fertilization, use of fungicides, crop rotation, and host genotype [4,16,17]. These data were not collected in the study and therefore no conclusions can be drawn on the correlation of the contamination rate and mycotoxin pattern on geographical and climatic conditions. However, the available meteorological data show that the annual average temperatures and precipitations were above the long-term average in all regions in both years. Furthermore, the incidence of mycotoxins in the samples from the Korça region seems lower than with samples from the other investigated western plain regions close to the Adriatic Sea. Considering that the level of agricultural development was similar in all investigated regions, the lower incidence can be explained by the climate differences between the temperate climate Korça and the typical Mediterranean climate regions of the western part of the country.

### 2.2. Comparison with Fusarium Mycotoxins Occurrence in Other Countries

Our result of the incidence of DON in wheat (23%) is comparable with the results presented by Griessler et al. [20], Škrbić et al. [37], Van Der Fels-Klerx et al. [41], and Juan et al. [43] (Table 5). Škrbić et al. [37] reported the incidence rate of 27.8% in the harvesting year 2007. A similar contamination rate was reported in Italy (28.1%) [43], Southern Europe (27.6%) [20], and in the sampling years before 2009 from Finland (29.9%), Norway (29.4%) and Sweden (20.6%) [41], while in other reports the incidence rates were higher i.e., 100% in the year 2011 in Norway [45], 96.7% in the year 2013 in Finland [18], 71.4% in the Netherlands [41], 46.5% in Poland [48], 74.1% in Catalonia, Spain [35], 95% in Navarra, Spain [38], 59.6% in Italy [46], 75% and 68.8% in Slovenia [34,47], 65% in Croatia [44], 85.7%–93.3% in Serbia [40] as well as 73.1% in Romania [42].

The maximum level of DON in the wheat commodity in our study was much lower than in a significant number of reports from other countries. The highest reported levels were 10,000 µg/kg in a sample from the Netherlands [41], 5865 µg/kg and 5510 µg/kg in samples from Finland [18,41], 3306 µg/kg in a sample from Serbia [40], and 3700 µg/kg [34] and 3070 µg/kg [47] in samples from Slovenia. However, in some studies from Poland, Croatia, Serbia, and Spain, respectively, the highest reported levels were at 100 µg/kg [48], 278 µg/kg [44], 309 µg/kg [37], and 437 µg/kg [35].

The incidence rate of DON in maize samples (24%) was comparable with incidence data for maize commodity from Slovenia (35.3%) [47] or Romania (42.9%) [49], but lower than the incidence rates of 71%–87.9% in this crop commodity in other studies reporting data from Slovenia [34], Spain [35]), Northern Europe [41] and Croatia [44] (Table 6). The highest determined level of DON (799 µg/kg) was comparable to the highest reported level in the study from Spain (580 µg/kg) [35], but lower than in all other studies in the range of 1269.9 to 14,420 µg/kg.

The incidence rate of ZEA in maize samples was 4.4%, similar to data from Romania (7.1%) [49], but lower than in all other studies where the occurrence rate of 13.3%–78% was given (Table 6). The highest determined level of ZEA in our study (263 µg/kg) was higher than the data reported by Manova and Mladenova [33] and Gagiu et al. [49], but much lower than in all other studies given in Table 6 (611–1000 µg/kg). The contamination rate of T-2 was similarly low, as in the reports of Jakovac-Strajn et al. [34] and Cano-Sancho et al. [35]; however, Pleadin et al. [44] reported a contamination rate of 57%.

The incidence of FBs (sum of FB1 and FB2) in our study (76%) was found to be similar with the published data on the incidence from Croatia (90%) [44] but was higher than in the other studies [34,47]. The highest determined level (16,970 µg/kg) is comparable to the concentration reported by Kirinčič et al. [47]. However, both values are much higher than those reported elsewhere [33,34,44].

## 3. Materials and Methods

### 3.1. Sample Collection

Samples were collected from different regions, taking into account the country’s geography and production. According to the Food and Agriculture Organization (FAO) of the United Nations database [51], which provides data relating to food and agriculture for countries worldwide, the yearly production of wheat, maize, barley, and rye in Albania is around 275,000 tonnes, 380,000 tonnes, 7500 tonnes, and 3000 tonnes, respectively. Correspondingly, mainly wheat and maize samples were collected, but also a few samples of barley and rye. Winter wheat and maize were sampled after their respective harvesting seasons from five main agriculture regions in Albania: Fieri, Lushnja, Kruja, Elbasan, and Korça. The sampling of wheat commodity in the regions of Fieri, Lushnja, Elbasan, and Kruja was carried out in June 2014 and June 2015, while in the Korça region it was performed in July 2014 and July 2015. Maize samples were collected in October 2014 and October 2015. The samples were taken from the warehouses. The weather conditions were not recorded at the time of sampling or during the growth of the grain. The sampling procedure was carried out according to the Commission Regulation (EC) No 401/2006 [53] to ensure representative samples. However, the study was performed as a research, not as a part of official control. Seventy-one wheat and 45 maize samples were collected in two harvesting seasons, 2014 and 2015. Specifically, 35 wheat and 31 maize samples belonged to the 2014 harvesting year, while 36 wheat and 14 maize samples were from 2015. In the year 2015, we were able to obtain the consent of only 14 farms for the maize samples to be taken. In addition, in Korça and Fieri, seven samples of barley and two samples of rye were collected (two samples of rye and five samples of barley in 2014, and two samples of barley in 2015).

### 3.2. Standards and Chemicals

Mixed trichothecene standard solution in acetonitrile (DON, 3-AcDON, 15-AcDON, T-2, HT-2, DAS, and NEO) produced by Trilogy (Washington, MO, USA) and single standards of ZEA, FB1, and FB2 (Romer Labs, Tulln, Austria) were used. Stock standard solutions and the mixed working standard solutions were prepared in acetonitrile and stored in amber glass vials at –20 °C. The concentrations of stock standard solutions were 100 µg/mL (DON, 3-AcDON, 15-AcDON, T-2, HT-2, DAS and NEO) and 50 µg/mL (ZEA, FB1, FB2). Acetonitrile, methanol, acetic acid (Sigma-Aldrich, Steinheim, Germany), and ammonium acetate (Merck, Darmstadt, Germany) were p.a. or LC–MS grade purity. Deionized water was prepared using a Milli-Q system (Millipore, Bedford, MA, USA).

### 3.3. Sample Preparation

For the simultaneous determination of mycotoxins (DON, 3-AcDON, 15-AcDON, ZEA, FB1, FB2, T-2, HT-2, DAS, and NEO), a procedure described in detail by Topi et al. [54] was used. The procedure consisting of the extraction of mycotoxins from ground cereal samples and liquid chromatography–tandem mass spectrometry (LC–MS/MS) was based on the analytical procedures of Rasmussen et al. [55], Lattanzio et al. [56] and Schenzel et al. [57]. Samples were ground to a particle size of 1 mm using a laboratory mill Retsch ZM 100 (Haan, Germany). Ten grams of a sample were shaken with 100 mL of an acetonitrile-deionised water mixture (84 + 16) for 1 h using an IKA HS 501 digital linear shaker (IKA Labortechnik, Staufen, Germany). A total of 4 mL of the filtered extract was evaporated under vacuum to dryness using a Syncore Polyvap system (Büchi, Flawil, Switzerland). For mycotoxin concentrations above the calibration range, the filtered extracts were diluted for further work. The dry residue was reconstituted in 0.5 mL of a methanol-deionised water mixture (20 + 80). An aliquot—10 μL of the solution—was injected into the UPLC–MS/MS system (Acquity UPLC H Class system) coupled with a triple-quadrupole mass spectrometer (Xevo TQ MS) equipped with an electrospray ionization (ESI) interface and MassLynx software for data collection and processing (Waters, Milford, MA, USA). The vials were kept in the autosampler at 15 °C. For the matrix-matched calibration, 4 mL portions of the filtered extracts were spiked with the appropriate amounts of standard solutions and prepared along the samples.

### 3.4. LC–MS/MS Analysis

For the LC–MS/MS determination, the conditions reported by Topi et al. [54] were applied. Chromatographic separation was performed on a Zorbax Eclipse Plus C18 Rapid Resolution HD column, 2.1 × 100 mm, 1.8 μm (Agilent, Santa Clara, CA, USA). The mobile phase consisted of two components mixed in gradient mode. Component A was deionized water and component B was methanol, both containing 0.5% acetic acid and 2.5 mM ammonium acetate. The starting composition of the eluent was 95% A and 5% B. The portion of component B was linearly increased to 40% within 4 min and further increased to 70% within the next 8 min. This latter composition was held for 4 min, and then component B was increased to 90% in 1.5 min. The proportion of component B was held at 90% for 2.5 min and then returned back to 5% in 1 min. The final composition was held for 4 min. The mobile phase flow rate was 0.3 mL/min, and the column temperature was 40 °C. MS/MS analysis was carried out in multiple reaction monitoring (MRM) mode switching between positive and negative ionisation mode during a single run. The capillary voltage was 3.4 kV (ESI+) and 3.0 kV (ESI−), the desolvation temperature was 500 °C, the ion source temperature was 150 °C and the collision cell voltage was 20 V. Specific MS/MS parameters related to determined mycotoxins (retention times, ionisation mode, and monitored transitions) are presented in Table 7.

### 3.5. Method Validation

The limit of detection (LOD) of the single analytes was determined at a signal-to-noise ratio of 3:1. A value 3.3 times the LOD was selected as the LOQ. The recoveries and precision were tested using maize and wheat samples spiked with *Fusarium* toxins at the concentration levels of 50, 100, and 500 µg/kg.

The linearity of the method was tested in the concentration range of 50–500 µg/kg using matrix-matched standard solutions analysed in triplicates. Good linearity was proven for all analytes with correlation coefficients higher than 0.997. The accepted limit of detection (LOD) and the limit of quantification (LOQ) of all single *Fusarium* toxins were 15 µg/kg and 50 µg/kg, respectively. The mean recoveries of single toxins determined in maize at the tested concentration levels were between 90% and 117%. The recoveries were between 87% and 112% in wheat, except for DON, which was 124%. The reproducibility expressed as RSD_R_ was less than 16% for all *Fusarium* toxins in maize and ≤30% for all *Fusarium* toxins in wheat. The reproducibility and mean recoveries of the toxins were in line with the criteria given in Commission Regulation (EC) No 401/2006 and its amendments [53] except the recovery of DON which slightly exceeded the required value.

## 4. Conclusions

In the study, the results of determination of ten *Fusarium* toxins in 125 samples from two seasons were obtained. They represent the very first insight into their occurrence in cereal commodities from Albania and a contribution to the knowledge on the issue in southern Europe.

Relevant *Fusarium* toxins in the region seem to be DON and FBs. Other toxins were detected in only a few samples (ZEA, T-2) or not at all (3-AcDON, 15-AcDON, HT-2, DAS, and NEO). The incidence was comparable with those reported in the neighbouring countries, but the FB concentrations in maize were significantly higher than reported elsewhere.

The incidence of mycotoxins in the samples from the Korça region seem lower than with the samples from the other investigated western plain regions close to the Adriatic Sea. Considering that the level of agricultural development is similar in all investigated regions, the lower incidence can be explained by the climate differences between temperate climate Korça and typical Mediterranean climate regions of the western part of the country.

A significant difference between the data from the years 2014 and 2015 indicates that data from further harvesting years need to be provided to adequately characterize the occurrence of *Fusarium* toxins in cereal grains in Albania. However, with regard to the incidence rates and the concentrations of DON and FBs, farmers should consider all principles of good agricultural practices including tillage, crop rotation, cultivar selection, planting date, irrigation and fertilisation regimes, insecticide/fungicide treatments, harvest timing, as well as drying, cleaning, segregation, and the storage of cereals under controlled conditions in order to reduce mycotoxin contamination and to ensure safe food and feed.

## Figures and Tables

**Table 1:** Occurrence of deoxynivalenol (DON) in the wheat samples from the harvesting seasons 2014 and 2015.

	2014	2015	2014–2015
No. of samples	35	36	71
No. of positive samples	12	4	16
Incidence of positive samples (%)	34	11	23
Mean (μg/kg)	540	657	569
Median (μg/kg)	512	257	477
Minimum (μg/kg)	112	198	112
Maximum (μg/kg)	919	1916	1916

**Table 2:** Occurrence of *Fusarium* toxins in maize samples from the harvesting seasons 2014 and 2015.

	DON	ZEA	FB1	FB2	FB1+FB2	T-2	Total
**2014**							
No. of samples	31	31	31	31	31	31	31
No. of positive samples	11	2	25	22	25	1	26
Incidence of positive samples (%)	35	6.5	81	71	81	3.2	84
Mean (μg/kg)	264	240	3460	2285	5470	106	
Median (μg/kg)	165	240	2694	1886	3669	106	
Minimum (μg/kg)	110	218	68.7	105	68.7	106	
Maximum (μg/kg)	799	263	9873	9218	16,970	106	
Toxin rate in positive samples (%)	42	7.7	96	85	96.3	3.8	
**2015**							
No. of samples	14	14	14	14	14	14	14
No. of positive samples	0	0	8	5	8	0	8
Incidence of positive samples (%)	0.0	0.0	57	36	57	0.0	57
Mean (μg/kg)	-	-	816	1573	1799	-	
Median (μg/kg)	-	-	308	479	389	-	
Minimum (μg/kg)	-	-	59.9	169	59.9	-	
Maximum (μg/kg)	-	-	3611	3836	6757	-	
Toxin rate in positive samples (%)	0.0	0.0	100	63	100	0.0	
**2014–2015**							
No. of samples	45	45	45	45	45	45	45
No. of positive samples	11	2	34	27	34	1	35
Incidence of positive samples (%)	24	4.4	76	60	76	2.2	78
Mean (μg/kg)	264	240	2819	2153	4445	106	
Median (μg/kg)	165	240	790	1564	1162	106	
Minimum (μg/kg)	110	218	59.9	105	59.9	106	
Maximum (μg/kg)	799	263	9873	9218	16,970	106	
Toxin rate in positive samples (%)	32	5.9	97	79	97	2.9	

DON: deoxynivalenol; ZEA: zearalenone; FB1: fumonisin B_1_; FB2: fumonisin B_2_.

**Table 3:** The occurrence of DON in the wheat samples from the harvesting seasons 2014 and 2015 according to the regions.

	Fieri	Lushnja	Elbasan	Korça
No. of samples	11	22	10	28
No. positive samples	4	7	4	1
Incidence of positive samples (%)	36	32	40	3.6
Mean (μg/kg)	756	320	853	427
Median (μg/kg)	804	279	684	427
Minimum (μg/kg)	518	112	128	427
Maximum (μg/kg)	898	548	1916	427

**Table 4:** Occurrence of DON and FBs (sum of FB1 and FB2) in maize samples from the harvesting seasons 2014 and 2015 for each region.

	Fieri			Lushnja			Kruja			Elbasan			Korça		
	DON	FBs	Total	DON	FBs	Total	DON	FBs	Total	DON	FBs	Total	DON	FBs	Total
No. of samples	7	7	7	10	10	10	11	11	11	6	6	6	11	11	11
No. positive samples	0	4	4	0	7	7	7	10	10	3	5	6	1	7	7
Incidence of positive samples (%)	0.0	57	57	0.0	70	70	64	91	91	50	83	100	9.1	64	64
Mean (μg/kg)	-	6511	-	-	5959	-	237	4894	-	361	1837	-	167	3813	-
Median (μg/kg)	-	6615	-	-	6205	-	149	3288	-	343	903	-	167	874	-
Minimum (μg/kg)	-	255	-	-	389	-	110	83.8	-	160	594	-	167	60	-
Maximum (μg/kg)	-	12,559	-	-	14,566	-	799	13,906	-	579	6117	-	167	16,967	-
No. of samples above EU max level	0	2	-	0	5	-	0	4	-	0	1	-	0	2	-
Samples above EU max level (%)	0	29	-	0	50	-	0	36	-	0	17	-	0	18	-

**Table 5:** Occurrence of DON in wheat from different studies.

Country	Year of Sampling	Method of Analysis	LOD/LOQ (μg/kg)	Number of Samples	Positive Sample Rate (%)	Mean (μg/kg)	Median (μg/kg)	Max (μg/kg)	Reference
Albania	2014–2015	LC–MS/MS	15/50	71	23	569 ^a^	477 ^a^	1916	This study
Finland	2000–2009	-	100/-	338	29.9	-	0	5865	[41]
Netherlands	1989–2009	-	100/-	940	71.4	-	220	10,000	
Norway	1990–2009	-	100/-	832	29.4	-	0	1552	
Sweden	1999–2009	-	100/-	554	20.6	-	0	890	
Norway	2011	LC–MS/MS	-	28	100	-	383	1400	[45]
Finland	2013	LC–MS/MS	1.3/3.9	30	96.7	866	-	5510	[18]
Poland	2014	UHPLC–HRMS	-/25	99	46.5	25–960 ^a^	25–694 ^a^	2975	[48]
Southern Europe	2005–2009	ELISA	-/250	29	27.6	275	602 ^a^	2232	[20]
Italy	2009–2010	HPLC–MS/MS	5/15	47	59.6	172 ^a^	-	1230	[46]
Italy	2012	LC–MS/MS	5/10	57	28.1	10.96 ^a^	-	99.6	[43]
Spain (Catalonia)	2008	GC–MS	-/41	27 ^b^	74.1	190 ^a^	157 ^a^	437	[35]
Spain (Navarra)	2007–2008	GC–MS	-/10	123	95	59.6 ^a^	21.4 ^a^	1111.3	[38]
Slovenia	2007–2008	GC–MS	50/100	20	75	849 ^a^	420 ^a^	3700	[34]
Slovenia	2008–2012	HPLC–UV	-/50	80 ^c^	68.8	477 ^a^	-	3070	[47]
Croatia	2011	ELISA	20.5/-	51	65	223 ^a^	-	278	[44]
Serbia	2005, 2007	ELISA	-/-	103	85.7–93.3	283–606 ^a^	-	1090–3306	[40]
Serbia	2007	LC–MS/MS	0.3/1	54	27.8	33	-	309	[37]
Western Romania	2010–2011	ELISA	110/220	52	19.2–73.1	763.6–2263 ^a^	-	1440–3390	[42]

^a^ only positive samples considered; ^b^ wheat flakes; ^c^ wheat and wheat products; LOD: limit of detection; LOQ: limit of quantification.

**Table 6:** Occurrence of *Fusarium* toxins in maize samples from different studies.

Country	Year of Sampling	Method of Analysis	LOD/LOQ (μg/kg)	Toxin	Number of Samples	Positive Sample Rate (%)	Mean (μg/kg)	Median (μg/kg)	Max (μg/kg)	Reference
Albania	2014–2015	LC–MS/MS	15/50	DON	45	24	264 ^a^	165 ^a^	799	This study
				ZEA	45	4.4	240 ^a^	240 ^a^	263	
				T-2	45	2.2	106 ^a^	106 ^a^	106	
				FB1+FB2	45	76	4445 ^a^	1162 ^a^	16,970	
Netherlands	1989–2009	-	100/-	DON	142	84.5	-	500	5000	[41]
			50/-	ZEA	147	37.4	-	0	1000	
Sweden	1999–2009	-	100/-	DON	5	40.0	-	187.5	420.0	
Spain (Catalonia)	2008	GC–MS	-/45	DON	65	75.4	109 ^a^	93 ^a^	580	[35]
			-/57	T-2	65	0	-	-	-	
			-/30	HT-2	65	6.2	41 ^a^	34 ^a^	65	
Slovenia	2007–2008	GC–MS	50/100	DON	58	87.9	1355 ^a^	480 ^a^	14,420	[34]
			20/50	ZEA	58	50.0	199 ^a^	180 ^a^	640	
			60/200	FB1+FB2	58	39.7	1336 ^a^	468 ^a^	6489	
			50/100	T-2	58	1.7	290 ^a^	290 ^a^	290	
			50/100	HT-2	58	1.7	2300 ^a^	2300 ^a^	2300	
Slovenia	2008–2012	HPLC–UV	-/50	DON	34 ^b^	35.3	1328 ^a^	-	11,800	[47]
			-/5	ZEA	34 ^b^	17.6	823 ^a^	-	4578	
			-/200	FB1+FB2	34 ^b^	23.5	4092 ^a^	-	27,483	
Croatia	2011	ELISA	20.5/-	DON	63	71	1565 ^a^	-	2942	[44]
			2.1/-	ZEA	63	78	187 ^a^	-	611	
			24.5/-	FB	63	90	1756 ^a^	-	4438	
			4.1/-	T-2	63	57	24 ^a^	-	42	
Serbia	2008–2015	ELISA	75/-	FB1+FB2	614	34.4–100	580–4310	<75–2590	41,440	[50]
Bulgaria	2007	HPLC	17.7/58.8	ZEA	19	21.1	80.6 ^a^	-	148.0	[33]
		LC–MS/MS	27.3/90.8	FB1+FB2	19	94.7	1150	-	4050	
Romania	2012–2015	ELISA	18.5	DON	91 ^c^	42.9	82.39	<18.5	1269.94	[49]
			1.75	ZEA	84 ^c^	7.1	1.92 ^a^	-	7.05	

^a^ only positive samples considered; ^b^ maize and maize products; ^c^ cereals and cereal-based food.

**Table 7:** Retention times of *Fusarium* toxins, ionization mode and monitored transitions.

Analyte	Ionization Mode	Retention Time (min)	Precursor Ion (*m*/*z*)	Quantifier Ion (*m*/*z*)	Qualifier Ion (*m*/*z*)
DON	ESI+	3.14	297.3	203.1	249.1
NEO	ESI+	4.10	400.3	185.1	305.2
3-AcDON	ESI+	5.00	339.1	203.1	137.0
15-AcDON	ESI+	5.00	339.1	136.9	261.1
DAS	ESI+	7.06	384.3	307.2	247.2
HT-2	ESI+	8.91	442.4	215.1	263.2
T-2	ESI+	10.27	484.4	185.1	215.2
ZEA	ESI-	11.30	317.2	131.0	174.9
FB1	ESI+	10.20	722.4	334.2	352.2
FB2	ESI+	12.70	706.4	318.2	336.2

DON: deoxynivaleol; NEO: neosolaniol; 3-AcDON: 3-acetyldeoxynivalenol; 15-AcDON: 15-acetyldeoxynivalenol; DAS: diacetoxyscirpenol; ZEA: zearalenone; FB1: fumonisin B_1_; FB2: fumonisin B_2_; ESI: electrospray ionization.

## Data Availability

Not available.

## Sample Availability

Not available.
